# Magnetic Domain Walls and Macroscopic Magnetization-Related Elastic and Anelastic Effects during Premartensitic Transition in Ni_2_MnGa

**DOI:** 10.3390/ma12030376

**Published:** 2019-01-25

**Authors:** Sergey Kustov, Jaume Rosselló, Miguel Lluís Corró, Vladimir Kaminskii, Konstantin Sapozhnikov, Andrey Saren, Aleksei Sozinov, Kari Ullakko

**Affiliations:** 1 Departament de Física, Universitat de les Illes Balears, 07122 Palma de Mallorca, Spain; j.rossello.coll@gmail.com (J.R.); mikelet9@hotmail.com (M.L.C.); 2 Faculty of Laser Photonics and Optoelectronics, ITMO University, 197101 St. Petersburg, Russia; kam-vladimiro@yandex.ru (V.K.); k.sapozhnikov@mail.ioffe.ru (K.S.); 3 Solid State Physics Division, Ioffe Institute, Russian Academy of Sciences, 194021 St. Petersburg, Russia; 4 Material Physics Laboratory, Lappeenranta University of Technology, FI-57170 Savonlinna, Finland; andrey.saren@lut.fi (A.S.); Oleksii.Sozinov@lut.fi (A.S.); kari.ullakko@lut.fi (K.U.)

**Keywords:** elasticity, anelasticity, eddy currents, Ni–Mn–Ga, premartensite

## Abstract

The temperature and field dependences of internal friction and Young’s modulus are studied using a high-resolution ultrasonic (90 kHz) technique in stoichiometric ferromagnetic Ni_2_MnGa shape memory alloy close to the premartensitic transformation temperature, *T_PM_*, in the demagnetized state and under moderate fields. Several new effects observed like an apparent Young´s modulus softening close to *T_PM_* under moderate fields, instead of the hardening outside this range, as well as existing controversies in the apparent elastic and anelastic properties of Ni_2_MnGa close to *T_PM_* are explained by microeddy and macroeddy current relaxations that to date have been disregarded.

## 1. Introduction

Ni–Mn–Ga are archetypal magnetic shape memory alloys with different sequences of magnetic and structural transitions that demonstrate a number of unique physical properties [1]. Structural transformations occur from the ordered cubic L2_1_ (or B2) austenite to non-modulated tetragonal or modulated monoclinic martensites (L1_0_, 10M, 14M) [2]. Near-stoichiometric Ni_2_MnGa alloys transform from the ferromagnetic L2_1_ cubic phase (C) into ferromagnetic martensite via weakly first-order intermediate transition at a temperature $T_{PM}$ into a premartensitic (PM) structure [3,4]. The latter is spatially inhomogeneous state with a nanometric characteristic length scale [5] and an overall cubic symmetry that is preserved. The origin of premartensite transition (PMT) and the structure of the PM phase remain subjects of intense discussion [5,6,7,8,9]. In fact, the properties and formation of such structures, which are spatially heterogeneous on the nanoscale, is a generic problem in ferroic and multiferroic systems [9,10], with ferroelastic incipient ferroelectric SrTiO_3_ with perovskite structure being an interesting example, see e.g., Ref. [10]. A crucial role of the ferroelastic domain structure and domain wall (DW) relaxation below the temperature of improper ferroelastic transition in SrTiO_3_ on elastic and anelastic properties during a number of widely discussed structural modifications has long been recognized [11]. In the case of PMT in ferromagnetic cubic Ni_2_MnGa, an important role of magnetoelastic coupling and lattice softening of L2_1_ ordered phase has traditionally been postulated, see e.g., [3], appealing for acoustic studies of PMT. Surprisingly, until recently, existing theoretical approaches to PMT and interpretations of acoustic experiments disregarded such important contributors to anelastic and apparent elastic effects as ferromagnetic DWs. Planes et al. [3] constructed a Landau-type model for the first-order PMT, and eventually related the change of elastic constants under a saturating field with variations of the value of macroscopic magnetization. However, the importance of variations of macroscopic magnetization clearly point to the role of magnetic domains and DWs in elastic softening during PMT rather than the role of the assumed dynamical lattice properties [3]. In a very recent work [12], resonant ultrasonic spectroscopy (RUS) was used to study effective shear modulus of polycrystalline Ni–Mn–Ga samples of different compositions. For the stoichiometric alloy, the elastic softening during ferromagnetic ordering and PMT was attributed exclusively to elastic strain coupling with two distinct order parameters. On the other hand, Seiner et al. [13] reported a very strong effect of antiphase boundaries and magnetic domain size and structure on elastic and anelastic phenomena during PMT. They emphasized the need to consider the role of magnetic domains in the magnetoelastic coupling of Ni_2_MnGa, but this idea has not received the attention it deserves.

Three canonical components of magnetomechanical internal friction (IF) and related modulus softening, known as the ΔE-effect, are considered [14,15,16]: two linear eddy current relaxations, microeddy and macroeddy, and the non-linear hysteretic term. The non-linear term and the fourth, recently discovered category, low-temperature relaxation due to the thermal freezing of DWs during re-entrant spin glass transition [16], are unrelated to the subject of the present study. The microeddy current relaxation is traditionally ascribed to the short-range (less or much less than the average domain size) reversible displacements of individual DWs, whereas the macroeddy one exists in partially macroscopically magnetized samples, and operates at a scale of the penetration depth of the electromagnetic wave, averaging over many domains [14,15,17]. All of the magnetomechanical effects vanish at saturation. A straightforward way to reveal DW-related microeddy current relaxation, as well the macroeddy one under non-saturating fields, is to verify the existence of a characteristic frequency dispersion of apparent elastic and anelastic properties. However, previous experimental studies of the elastic and anelastic phenomena in near-stoichiometric Ni–Mn–Ga alloys around PMT [3,6,12,18,19,20,21,22,23,24,25] used various techniques and distinct parameters were derived, impeding their direct quantitative comparison. Elastic constants *C*_11_, *C*_12_, *C*_44_, or *C*′ (and hence the Young’s modulus along the [100] direction, since $E_{100}\approx 3C\prime$ in the cubic Ni_2_MnGa) were determined by pulse-echo and transmission ultrasonic techniques [3,6,18,19,20], *C*′ [6,13], some effective shear modulus in polycrystals [12,21] was determined by RUS, while the storage modulus was determined in dynamical mechanical analyzers (DMA) [22,23,24,25]. The scatter of experimental data on elastic softening during PMT is exemplified by data for *C*_44_, covering the range from 8–10% [18,19,20] to virtually not existing at a rather high frequency of 20 MHz [6].

Another crucial factor that potentially reveals eddy current relaxations is the frequency dependence of the effect of the saturating magnetic field on the apparent elastic constants. This effect is inherent in Ni_2_MnGa, and was conventionally attributed to the change of the lattice dynamics, see e.g., [1]. However, analysis of the available data shows a strong frequency dispersion of the effect of the saturating field on *C*′ for the frequencies 10^2^–10^4^ kHz, which ischaracteristic for microeddy current relaxations. Gonzàles-Comas et al. [26] found by pulse-echo technique a 1.5% increase of *C*′ under the saturating field in the cubic phase of Ni_2_MnGa, whereas a twofold and 40% increase was observed using RUS by Seiner et al. [6,13] in the cubic phase and during PMT, respectively, for a quenched Ni_2_MnGa crystal. In agreement with the difference between the effects of the saturating field on low-frequency and high-frequency elastic softening, Recarte et al. found that *C*′ hardening by the saturating field in Ni–Mn–Ga, which was estimated from inelastic neutron scattering, does not exceed 12% [27], which is well below the low-frequency (kHz range) effects [6,13]. Moreover, in other studies, variations of TA_2_ energy under the saturating field remained within the experimental error in Ni–Mn–Ga [28] and negligible in Ni–Fe–Ga [29].

The interpretation of the IF peak during the C↔PM transition remains also controversial: critical slowing down during the second order transition [12], additional anelastic strains and “criticality” close to the PMT [6], localized soft modes [19], and the co-existence of phases during first-order phase transition [22,23,24,25], not involving transitory term at infrasonic frequencies [23,24,25]. The influence of the applied field on IF at $T_{PM}$, to the best of our knowledge, has not been studied.

We have shown recently [30] that enhanced apparent elastic softening and IF below *T_C_* in the cubic Ni_2_MnGa stem from microeddy and macroeddy current relaxations operating not at the atomic scale, but at the scale of magnetic domains and even the sample size, respectively. This interpretation explains the frequency dependence of apparent “elastic” softening and the possible difference between the effect of the magnetic field on the apparent elastic properties and the phonon energies. Here, we show that this new approach, involving eddy current relaxations, accounts for existing and new, as reported in the present work, details of the elastic and anelastic properties of Ni_2_MnGa during PMT.

## 2. Materials and Methods

The Ni_2_MnGa crystal that was studied was grown at AdaptaMat Ltd. using directional solidification. The crystal was homogenized for 96 h at 1300 K and slowly cooled (50 K/h), ensuring Heusler-type atomic ordering and minimizing defect formation. The specimen 1 × 1.1 × 7 mm^3^ was spark cut along the [100] direction, ground, and electropolished. The phase transformation temperatures were derived from the resistivity and magnetic permeability. The alloy showed $T_{C}$ ≈ 383 K, $T_{PM}$ ≈ 261 K, and the start temperatures of the direct and reverse martensitic transformations $M_{s}$ = 201 and $A_{s}$ = 206 K, respectively. The temperature dependences of the IF and Young’s modulus (YM) at ~90 kHz were studied using the piezoelectric composite oscillator technique [31]. A home-made experimental setup [32,33] permitted determining the logarithmic decrement, *δ*, and resonant frequency, f, of the fundamental mode of the longitudinal oscillations of the sample for temperatures between 80–400 K.

The logarithmic decrement of the sample δ was conventionally derived [31,32,33] from the total decrement of the composite oscillator, $\delta_{c}$, and the decrement of the quartz transducer alone, $\delta_{q}$, using a kind of rule of mixture equation:

(1) $$\delta_{c}m_{c}=\delta m_{s}+\delta_{q}m_{q},$$

with $m_{q}$, $m_{s}$ and $m_{c}=m_{q}+m_{s}$ representing the mass of the quartz transducer alone, the mass of the sample, and the total mass of the composite oscillator, respectively.

The effective Young’s modulus (YM), E, was calculated from the resonant frequency, the density *ρ*, and the length *L* of the sample, as $E=4\rho f^{2}L^{2}$. The resonant frequency of the sample f was determined solving the equation [34,35]:

(2) $$m_{q}f_{q}\tan\frac{\pi f_{c}}{f_{q}}+m_{s}f\tan\frac{\pi f_{c}}{f}=0,$$

where *f_c_* and *f_q_* are respectively the resonant frequencies of the composite oscillator and the quartz transducer alone. Equation (2) was used instead of the approximate one [31], since the YM variations close to *T_PM_* were substantial. The measurements could be performed under longitudinal magnetic fields of up to *H* = 18 kA/m. The magnetic field was created by a 400-mm long, 60-mm diameter solenoid. The homogeneity of the applied field in a working space containing an oscillator was better than 0.5%. The homogeneity of the true magnetic field inside the rectangular bar-shaped sample was, of course, deteriorated by demagnetizing effects, and the values of the true field are difficult to compute. However, using a rather thin sample polarized in the axial direction allowed us to keep the demagnetizing factor rather small. An approximation to estimate the demagnetizing factor is to consider the sample as an ellipsoid with axial ratios of 7:1.1:1.0. Then, for the field applied along the sample, the demagnetizing factor is around 0.04 [36]. In our experiments, an attempt was undertaken to employ a longer sample that measured two ultrasonic half waves, oscillating in the second harmonic, in order to further reduce the demagnetizing factor to ca. 0.015. Unfortunately, the peak values of the total internal friction of the oscillator $\delta_{c}$, Equation (1), with a longer (heavier) sample were too high to be measured during the PMT when the polarized field was applied.

The total of three specimens was tested in order to choose the appropriate harmonic and length of the sample. All of the samples showed qualitatively the same major effects reported below (“softening” under the non-saturating field during the PMT, splitting of the IF peak and of the YM minimum around $T_{PM}$, and substantial IF hysteresis in the cubic phase after cooling the sample below $T_{PM}$). Detailed studies under polarizing fields were performed for one of the samples, with the length minimizing the maximum value of the mismatch between the resonant frequencies of the sample and the quartz transducer over the temperature range studied.

A low oscillatory strain amplitude, 2 × 10^−7^, was stabilized in the experiments to discard the non-linear anelastic effects.

## 3. Results

**Figure 1:**
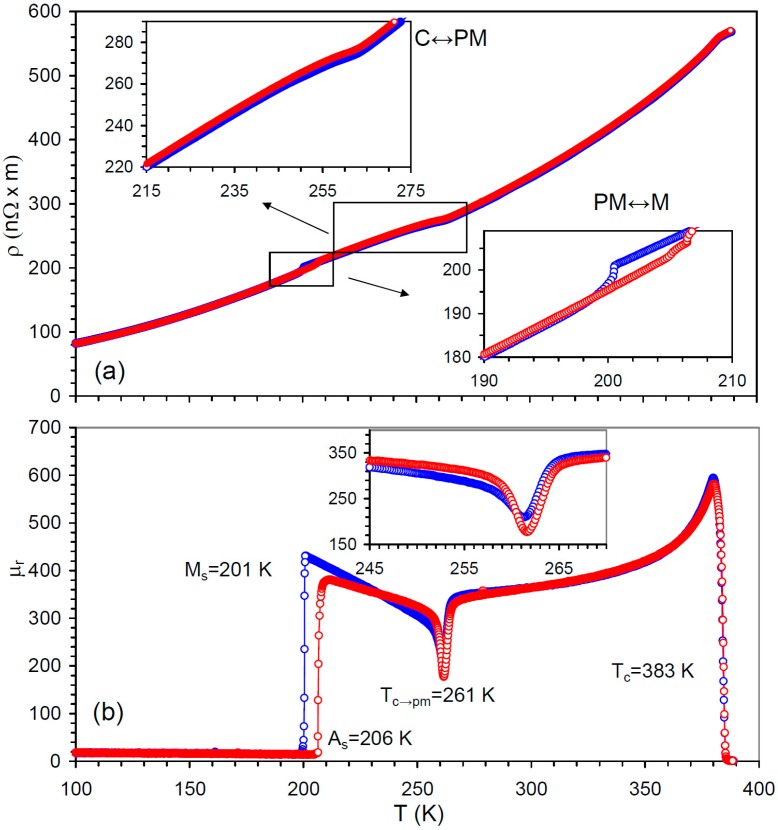
Temperature dependence during cooling (blue symbols) and heating (red symbols) of (**a**) resistivity, ρ, (**b**) reversible permeability $\mu_{r}$, for a single crystalline sample of stoichiometric Ni_2_MnGa. The insets in (**a**) show details of the resistivity behavior during the cubic–premartensite (C↔PM) and premartensite–martensite (PM↔M) transformations. The Curie temperature *T_C_* = 383 K, the temperature of the maximum rate of the C→PM transition, *T_C-PM_* = 261 K, and the temperatures of the start of the direct, Ms, and reverse, As, martensitic transformations are indicated in (**b**). The inset in (**b**) shows details of the permeability minimum during C↔PM transformation.

Figure 1 shows the resistivity, ρ, and reversible permeability, $\mu_{r}$, versus temperature over the range covering the relevant phase transformations: para-ferro, C↔PM and premartensitic–martensitic. ρ and $\mu_{r}$ were derived from the real and imaginary parts of the alternating current (AC) impedance [37]. The abrupt initiation and narrow range of the ferromagnetic ordering together with a very sharp and intense permeability drop during C↔PM transition, Figure 1b, prove the high homogeneity of the sample. The C↔PM hysteresis in our high-quality samples is around 1 K, as shown in the inset in Figure 1b.

**Figure 2:**
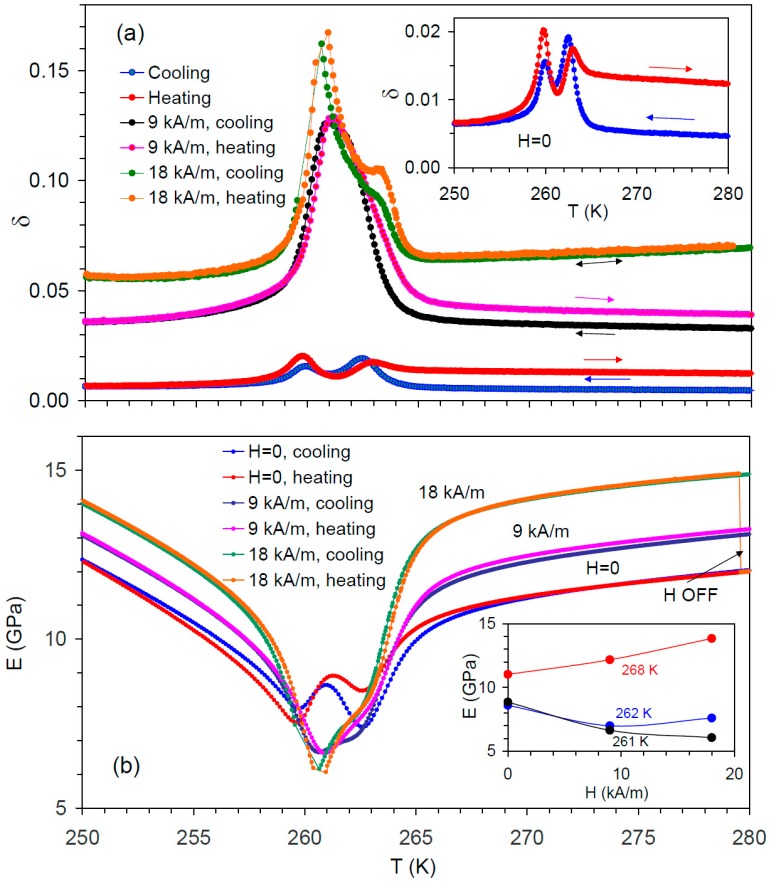
Temperature spectra of (**a**) internal friction, δ, and (**b**) Young’s modulus, *E*, of a single crystalline Ni_2_MnGa sample during cooling and heating in a demagnetized state, *H* = 0, and under an applied field of 9 kA/m and 18 kA/m. The inset in (**a**) shows details of the internal friction spectra for the demagnetized state on an expanded scale. The inset in (**b**) shows the field dependences of the Young’s modulus at 268 K, 262 K, and 261 K. The arrow H OFF in (**b**) shows the abrupt drop of the Young’s modulus down to the values of the demagnetized state after switching off magnetic field *H* = 18 kA/m during the heating scan.

Figure 2a,b shows the IF and YM versus temperature for the demagnetized state of the sample and under moderate (below saturation) fields. Since $E_{100}\approx 3C\prime$ in cubic Ni_2_MnGa, the values of the effective YM in the demagnetized state are in good agreement with previous data on C′, as obtained by RUS over the same temperature and frequency ranges [6,13,38]. A double IF maximum and corresponding double YM minimum are observed over the PMT range for the demagnetized state. Similar IF peak splitting during PMT was reported by Seiner et al. [13]. A double IF maximum and double longitudinal velocity minimum (not commented upon) can also be found in the data by Stenger and Trivisonno [19]. The IF shows the hysteresis of the PMT ca. (0.5–1.0) K, which is consistent with the permeability data, as shown in Figure 1b. The IF in the cubic phase is notably higher during heating from below $T_{PM}$ than in the demagnetized state (cooling from above $T_{C}$), inset in Figure 2a. The “demagnetized” low IF level is recovered after heating the sample beyond $T_{C}$; the IF hysteresis is reproduced in consecutive thermal cycles.

Measurements under moderate fields reveal several new effects during PMT. Firstly, the YM, instead of hardening under a saturating field [6,13], shows a substantial decline around PMT, as shown in Figure 2b. The effect is opposite in the cubic and premartensitic phases: the YM increases under a moderate field, as shown in Figure 2b. This “softening” instead of “hardening” that is observed in the cubic phase and under saturating fields cannot be related to the certain heterogeneity of the true field inside the sample due to demagnetizing effects. Indeed, conventional “hardening”, monotonous versus field, cannot provoke the decrease of the elastic constants, whatever the true field distribution. Secondly, the IF increases with *H* in the premartensitic phase and during PMT, which is similar to the effect found in the cubic phase [30]. However, the IF that increases under the field is much more pronounced during PMT than in the cubic and premartensitic phases, as shown in Figure 2a.

The cooling–heating rate of 0.5 K/min, and oscillatory strain amplitude of 2 × 10^−7^.

## 4. Discussion

According to [30], the additional intense softening of elastic constants in Ni_2_MnGa below $T_{C}$ in the demagnetized state and under moderate fields (below saturation) is not elastic, but rather a relaxational effect that is related to classical linear microeddy and macroeddy current relaxations. In the cubic phase, the IF measured at *f* ~ 10^5^ Hz increases under the non-saturating field due to the net magnetization of the sample, producing a macroeddy current IF. On the other hand, the YM hardens under the field due to the suppression of the microeddy current ΔE-effect: for f ~ 10^5^ Hz, the latter is predominant in the cubic Ni_2_MnGa over the macroeddy ΔE-effect [30] due to the following relation between f and the frequencies of microeddy and macroeddy relaxations, $f_{\mu }$ and $f_{M}$, respectively:

(3) $$f_{M}<<f<<f_{\mu },$$

Below, we analyze, using the same concept of eddy current relaxations, new features of elasticity and anelasticity during the PMT observed in the present work, and some as yet unexplained phenomena such as the weaker effect of the saturating field on C′ softening during PMT than in the cubic phase [13] or low-field magnetization hysteresis [8].

### 4.1. Brief Background: Microeddy and Macroeddy Current Relaxation Strength and Frequency

The microeddy and macroeddy ΔE-effect, $\left(\frac{\Delta E}{E}\right)$, and corresponding IF components, δ, are related to relaxation strength Δ:

(4) $${\left(\frac{\Delta E}{E}\right)}_{M,\mu }=\Delta_{M,\mu }^{}\frac{1}{1+{\left(f/f_{M,\mu }\right)}^{2}},$$

(5) $$\delta_{M,\mu }=\pi \Delta_{M,\mu }^{}\frac{f/f_{M,\mu }}{1+{\left(f/f_{M,\mu }\right)}^{2}},$$

where indices *M* and μ denote the macroeddy and microeddy components, respectively.

In the case of macroeddy relaxation, Equations (4) and (5) represent the first, most important component of the sum of a series [39], as discussed in [30]. As before [30], we use a solution for the longitudinal oscillations of a circular rod of radius *a* [40] as a rough estimate of $f_{M}$:

(6) $$f_{M}\approx \frac{\rho }{2\pi \mu_{0}\mu_{r}a^{2}},$$

where $\mu_{0}$ is the permeability of free space. Macroeddy relaxation occurs in the volume of a sample that is controlled by such macroscopic parameters as magnetic skin depth [15,17], and depends on such extrinsic parameters as sample size, as shown in Equation (6).

Macroeddy relaxation strength $\Delta_{M}^{}$ depends on the differential inverse magnetostriction ${\left(\partial B/\partial \sigma \right)}_{H}$ [15,39]:

(7) $$\Delta_{M}^{}=\frac{E_{U}}{\mu_{0}\mu_{r}}{\left(\partial B/\partial \sigma \right)}_{H}^{2},$$

where $E_{U}$ represents the unrelaxed YM. $\Delta_{M}=0$ in the demagnetized state and at saturation, when ${\left(\partial B/\partial \sigma \right)}_{H}=0$, and reaches a maximum at the intermediate applied fields that are rather close to saturation [14,39,41].

$f_{\mu }$ is evaluated from a relation that is similar to Equation (6), with the sample dimension *a* substituted for the characteristic magnetic domain size *l* [14,15,42]:

(8) $$f_{\mu }\approx \frac{\rho }{2\pi \mu_{0}\mu_{r}l^{2}}.$$

The strength of the microeddy current relaxation is [14,15,42]:

(9) $$\Delta_{\mu }^{}=A\frac{\mu_{0}\mu_{i}\lambda_{s}E_{U}}{I_{s}},$$

where *A* represents a numerical factor, $\mu_{i}\approx \mu_{r}$ represents the initial permeability, $\lambda_{s}$ represents the saturation magnetostriction, and $I_{S}$ represents the spontaneous magnetization. $\Delta_{\mu }$ is the highest in the demagnetized state and falls off to zero at saturation [14,15,16].

### 4.2. Parameters Affecting Microeddy and Macroeddy Current Relaxations during Premartensitic Transition in Ni_2_MnGa

Equation (6) to Equation (9) are sufficient to explain the details of the IF and apparent YM spectra around the PMT in the demagnetized state and under applied field below saturation. $\lambda_{s}$, $\mu_{r}$, and $E_{U}$ are the parameters controlling relaxations and suffering strong variations during the PMT. The absolute value of magnetostriction increases more than three times [6,43], whereas the permeability drops two times, Figure 1b. We could not determine $E_{U}$ directly, since the applied field was well below saturation in our experiments. However, the $E_{U}$ softening at $T_{PM}$ can be evaluated from C′ data under the saturating field [13] as approximately 40%.

Unfortunately, the numerical values of certain parameters that are involved in microeddy and macroeddy current relaxations remain unknown, making it impossible to calculate the absolute values of the internal friction, the ΔE-effect, and their temperature spectra. More specifically, the lack of knowledge of the values and the effects of the temperature and magnetic field on differential inverse magnetostriction, ${\left(\partial B/\partial \sigma \right)}_{H}$, impedes the calculation of the macroeddy current relaxation strength, as indicated in Equation (7), and, hence, the macroeddy current ΔE-effect, as indicated in Equation (4), and the damping effect, as indicated in Equation (5). As for the microeddy current relaxation, some important information that is missing is the effect of the temperature and magnetic field on the magnetic domain structure: the domain size controls the microeddy current-related effects through the relaxation frequency, as indicated in Equation (8). Below, we analyze qualitatively and, if possible, semi-quantitatively, the influence of $\lambda_{s}$, $\mu_{r}$, and $E_{U}$ variations on the frequencies and strengths of eddy current relaxations during the PMT.

#### 4.2.1. Eddy Current Relaxation Frequencies during Premartensitic Transition

The twofold sharp decrease of $\mu_{r}$ at $T_{PM}$ raises the frequencies of both microeddy and macroeddy relaxations, as shown in Equations (6) and (8). However, we argue below that after cooling below PMT $f_{\mu }$ actually decreases, which is likely due to the variation of domain size, Equation (8). The latter effect overcompensates for the decline of $\mu_{r}$ in $f_{\mu }$. Therefore, eventually, $f_{M}$ increases sharply around $T_{PM}$ but $f_{\mu }$ slightly decreases. Since $f_{M}<f<f_{\mu }$, both shifts promote corresponding IF components, Equation (5), more notably $\delta_{M}$.

#### 4.2.2. Eddy Current Relaxation Strengths during Premartensitic Transition

**Figure 3:**
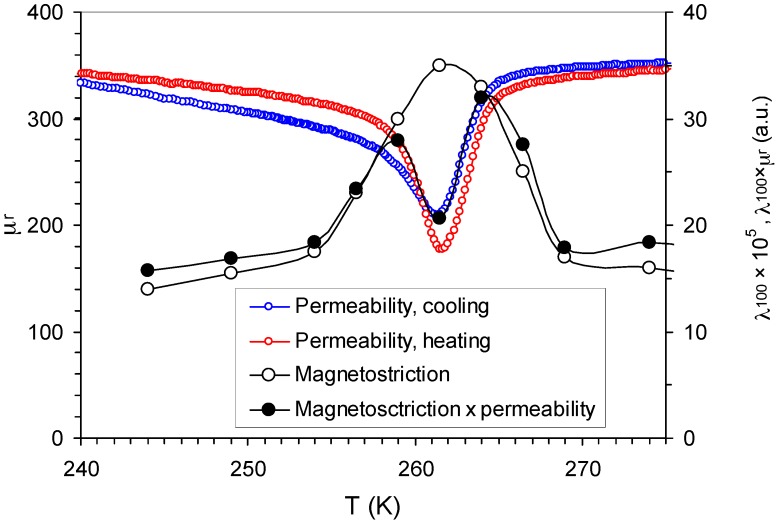
Temperature spectra of reversible permeability $\mu_{r}$, saturation magnetostriction $\lambda_{100}$ (values are taken from Figure 1b of Ref. [6]) and of their product $\lambda_{100}\times \mu_{r}$, which is proportional to the microeddy relaxation strength $\Delta_{\mu }$.

The $E_{U}$ softening tends to reduce both $\Delta_{\mu }$ and $\Delta_{M}$, as shown in Equations (7) and (9), by ca. 40% at $T_{PM}$. For the microeddy current relaxation, two other important parameters are initial permeability and saturation magnetostriction: microeddy relaxation strength is proportional to $\mu_{i}\approx \mu_{r}$ and $\lambda_{s}$, as shown in Equation (9). Variations of these two parameters during PMT are opposite: $\mu_{r}$ drops sharply by ca. 50%, as shown in Figure 1b, whereas the absolute value of $\lambda_{s}$ increases nearly three times [6,43]. Figure 3 visualizes the overall effect of these two competing parameters: it shows the temperature spectra of $\mu_{r}$ and magnetostriction in [100] direction $\lambda_{100}$ ($\lambda_{100}$ values are taken from [6]) and of their product, which is proportional to $\Delta_{\mu }$. A stronger increase of $\lambda_{100}$.

Dominates the drop of $\mu_{r}$, provoking an overall moderate increase of $\mu_{r}\times \lambda_{100}$. However, the dip in $\mu_{r}$ is very sharp at $T_{PM}$, and results in an abrupt local decline of $\mu_{r}\times \lambda_{100}$. To obtain $\Delta_{\mu }$ vs. *T*, the product $\mu_{r}\times \lambda_{100}$ must be scaled with $E_{U}$, as shown in Equation (9). The $E_{U}$ decline further reduces the microeddy relaxation strength at $T_{PM}$ by ca. 40%. Finally, $\Delta_{\mu }$ at $T_{PM}$ might become even lower than in the cubic phase.

$\Delta_{M}$ is controlled by the differential inverse magnetostriction ${\left(\partial B/\partial \sigma \right)}_{H}$ and permeability $\mu_{r}$, as shown in Equation (7). In contrast to $\Delta_{\mu }$, the decline of $\mu_{r}$ during PMT promotes macroeddy relaxation. The behavior of ${\left(\partial B/\partial \sigma \right)}_{H}$ has not been studied in the present work. Nevertheless, ${\left(\partial B/\partial \sigma \right)}_{H}$ is expected to increase with $\lambda_{s}$ if the possible increase of the saturating field at $T_{PM}$ is less than that of the $\lambda_{s}$, which is very likely, since $\lambda_{s}$ increases very strongly (more than3 times). Therefore, in contrast to $\Delta_{\mu }$, $\Delta_{M}$ increases notably during the PMT.

### 4.3. Interpretation of Elastic and Anelastic Effects during Premartensitic Transition Associated with Eddy Current Relaxations

Demagnetized state (microeddy current relaxation).

First, the local $\Delta_{\mu }$ decline at PMT, as shown in Figure 3, splits the microeddy IF maximum and YM minimum at $T_{PM}$, which is in full agreement with the experimental results, as shown in Figure 2 and [13,19].

Second, the overall moderate $\Delta_{\mu }$ decrease around $T_{PM}$ is the reason for the different effect of saturating field on elastic constants in the cubic phase and during the PMT. Indeed, if Equation (3) holds, Equation (4) yields:

(10) $${\left(\frac{\Delta E}{E}\right)}_{\mu }\approx \Delta_{\mu }^{}.$$

Since the saturating field eliminates all of the microeddy current relaxation-related effects, the decrease of the $\Delta_{\mu }$ at $T_{PM}$ implies a lower ΔE-effect, as shown in Equation (10), and less intense C′ and YM hardening under saturating field around $T_{PM}$ than in the cubic phase, which is in full agreement with the experimental observations [6,13]. As for $\delta_{\mu }$, Equations (3) and (5) yield:

(11) $$\delta_{\mu }\approx \pi \Delta_{\mu }\frac{f}{f_{\mu }}.$$

Equation (11) shows that the effect of $\Delta_{\mu }$ decreasing at $T_{PM}$ is partially compensated in $\delta_{\mu }$ by lower $f_{\mu }$ values during and after the PMT. Finally, depending on the specific values of several of the above-mentioned parameters, $\mu_{i}$, $\lambda_{s}$, $E_{U}$, domain size, and the overall level of $\delta_{\mu }$ at $T_{PM}$ can be both higher and lower than in the cubic phase. We mention here that Figure 2a shows the total IF, which includes a phase transition term, and is not related to eddy current relaxations. This term is not known since, to the best of our knowledge, the ultrasonic data on IF at $T_{PM}$ under the saturating field are not available. The low-frequency DMA data [23,24,25] do not include the relaxational microeddy component, either. However, high oscillatory strain amplitudes around 10^−4^ are typically employed in DMA tests [23,24], and DMA data around $T_{PM}$ predominantly represent the non-linear IF contribution [23].

Third, the microeddy eddy current origin of the IF in the cubic phase [30] allows one to interpret a new observation of the quasi-irreversible hysteresis of the IF in the cubic phase after cooling the sample below $T_{PM}$. This effect is consistent with the so-far unexplained low-field magnetization hysteresis in the cubic phase [8]. For the experiments performed at a frequency well below the microeddy relaxation frequency, $f<<f_{\mu }$, the increase of $\delta_{\mu }$ without concomitant YM softening during heating from below $T_{PM}$, Figure 2a, is a hallmark of DW microeddy relaxation, as shown in Equations (4) and (5). A more than twofold increase of $\delta_{\mu }$ after crossing $T_{PM}$ then presumably indicates that the corresponding decrease of the microeddy relaxation frequency $f_{\mu }$, as shown in Equation (5), is due to the increase of the characteristic magnetic domain size, as shown in Equation (8). Therefore, the absence of any detectable difference in the crystallographic structure accompanying magnetization hysteresis [8] is of no surprise. However, experimental confirmation of this prediction is pending. Both the IF hysteresis in the cubic phase and splitting of the IF peak and YM minimum at $T_{PM}$ decline under moderate fields, as indicated in Figure 2, due to the suppression of $\delta_{\mu }$ and ${\left(\frac{\Delta E}{E}\right)}_{\mu }$ by applied field. In addition, applied field overtakes the control of the domain structure, thus reducing $\delta_{\mu }$ hysteresis.

Internal friction and Young’s modulus under moderate field (macroeddy relaxation).

The most important factor affecting macroeddy current relaxation close to the PMT is a strong increase of $\Delta_{M}$, as discussed in Section 4.2.2. This increase explains the inversion of the effect of the moderate field on YM during the PMT and a stronger IF rise under applied field than that in the cubic phase. In the cubic phase, the microeddy component of the ΔE-effect, which is suppressed by the applied field, is predominant over the macroeddy one at f ~ 10^5^ Hz, hiding the characteristic YM minimum due to macroeddy current relaxation; this competition between the microeddy and macroeddy current ΔE-effect results in an “anomalous” simultaneous increase of the IF and effective YM within the non-saturating field [30]. The higher macroeddy relaxation strength around PMT promotes the macroeddy ΔE-effect and thus results in the “normal” macroeddy relaxation behavior: a YM minimum versus field concomitant with IF maximum [39]. These YM and IF extrema are normally observed at applied fields close to saturating ones [39,41], which could not be reached in the present study. Nevertheless, the IF maximum versus field was observed in the ferromagnetic cubic phase of Ni_2_MnGa not too far from $T_{C}$ [30], which is when the saturating field declines strongly.

## 5. Conclusions

A new interpretation of a number of elastic and anelastic phenomena during premartensitic transition in Ni_2_MnGa is suggested based on the concepts of microeddy and macroeddy current relaxations, which are associated respectively with the short-range oscillatory motion of ferromagnetic domain walls and stress-induced variations of the macroscopic magnetization of the sample. The approach used is unified with the description of additional “elastic” softening in the cubic Ni_2_MnGa below the Curie temperature.
